# Design, development and enhanced efficacy of host-derived antiviral peptides to prevent herpes simplex virus type 1 infection

**DOI:** 10.1007/s00726-026-03552-y

**Published:** 2026-08-13

**Authors:** Sanskruti Shrenik Patil, Rutwik Nilesh Garge, Anantha Padmanabha Aithal, Kishore Srinivasan, Rakesh Rahangdale, Raghu Chandrashekar Hariharapura

**Affiliations:** Department of Pharmaceutical Biotechnology, Manipal College of Pharmaceutical Sciences, Manipal Academy of Higher Education, Manipal, India

**Keywords:** Herpes simplex virus, Glycoprotein gD, Herpes virus entry mediator (HVEM), Therapeutic peptide, Residue scanning, Antiviral peptide

## Abstract

Herpes simplex virus (HSV) remains a pervasive global health concern and has the ability to challenge current therapeutic strategies because of its frequent recurrence and drug resistance. This research aimed to disrupt the critical interaction between the viral glycoprotein gD and the host cell receptor of herpes simplex virus, thereby blocking viral infection as a novel antiviral approach. An HVEM-derived native peptide NP (KEACGELTGTVCEP) targeting HSV was rationally designed, with no cytotoxicity and approximately 50% antiviral protection in vitro. Through computational residue scanning, docking and molecular dynamic simulation, seven modified variations of peptides were created to increase the potency and stability, which revealed that the modified peptide F4 with improved binding affinity docking. In vitro tests indicated its dose-dependent antiviral activity, and 60.58% protection at 250 µg/ml was achieved without toxicity. Although relatively less effective than Acyclovir, F4 (RQACGELTGTVCEP) has promising therapeutic potential. This integrated approach highlights the viability of Optimized HVEM-derived peptides as promising candidates with demonstrated antiviral efficacy, offering a strong foundation for future anti-HSV drug development.

**Supplementary Information:**

The online version contains supplementary material available at https://doi.org/10.1007/s00726-026-03552-y.

##  Introduction

Herpes simplex virus (HSV), a double-stranded DNA virus that belongs to the Herpesviridae family, is highly contagious and can cause lifelong infection. In 2025, an estimated 3.8 billion people under the age 50 globally are infected with HSV-1, and 520 million of age 15–49 are infected with HSV-2, according to the World Health Organisation (https://www.who.int/news-room/fact-sheets/detail/herpes-simplex-virus 2025; Harfouche et al. 2025). Each year, approximately 776,000 new cases occur, with a neonatal incidence of 5.3 per 10,000 births reported (Cole 2020; Cairns and Connolly 2021), which poses a significant public health burden. The disease contributes to 10–20% of all viral encephalitis cases worldwide (Levitz 1998; Whitley and Baines 2018), with the virus disseminated through direct contact with herpetic lesions, mucosal surfaces, vaginal fluids, and oral secretions, with an incubation period of 2–12 days. HSV-1 is typically acquired during childhood through oral contact and can cause oral and ocular infections, including labialis, stomatitis, and keratitis. The infection results in mucocutaneous vesicular lesions of the tongue, lips, gingiva, buccal mucosa, and palate. In contrast, HSV-2 is transmitted predominantly sexually and is responsible for genital herpes.

Epidemiological studies across India are sparse, highlighting regional variations in disease incidence. In 2020, two-thirds of the global population was aged 0–49 years, and nearly 4 billion people were seropositive for HSV-1, primarily through oral infections acquired during childhood, with more than 120 million new infections during the same year (Mukherjee et al. 2026). Globally, the oral HSV-1 seroprevalence was 58.6% among this age group, equating to approximately 3.4 billion individuals, whereas the genital HSV-1 seroprevalence among those aged 15–49 years was 10.2%, affecting 376 million people, reflecting an epidemiological shift toward greater sexual transmission in adolescence and adulthood. The African region had the highest oral HSV-1 seroprevalence, whereas the Western Pacific had the most infected individuals overall, with prevalence estimates calibrated from comprehensive regional systematic reviews despite data limitations, particularly for children. CDC clinical updates from 2024 indicate that HSV-1 is now a leading cause of first-episode genital herpes in young adults, attributed to declining childhood oral acquisition (leading to a lack of cross-protective immunity) and changes in sexual behavior. Recent studies in 2025 and 2026 show a high burden, particularly in clinical settings (Hufstetler et al. 2024). The overall HSV-1&2 seropositivity was 46.0%. Notably, 50.6% of women in the reproductive age group 18–30 were seropositive(Deka et al. 2025). In a 2026 study in South Asia of patients with genital ulcer disease (GUD), 18.6% of cases were caused by HSV-1(Mukherjee et al. 2026). A hospital-based study in Mysore reported an HSV-2 seroprevalence of 11.3% among 898 women(Madhivanan et al. 2007; AlMukdad et al. 2023). In Delhi, it was 23.3%, whereas community-based studies reported lower rates of 7–8% (Rathore et al. 2010), and among pregnant women, it was 8.7% in Northeast India (Biswas et al. 2011). Additionally, an investigation in Uttar Pradesh revealed that 9.3% of 1,578 acute encephalitis syndrome cases were HSV-related, with HSV encephalitis showing the highest rates of residual neuropsychiatric disability(Jain et al. 2014).

The virus consists of double-stranded DNA enclosed in a capsid, which is then covered by tegument proteins and an envelope (Figure S1). The members of this genus are characterised by a broad host range, rapid replication cycle, cytopathic effects, and the capacity to establish latency (Riley 1998). (Heath and Dembowski 2022). The viral envelope consists of 12 glycoproteins, namely, gB, gC, gD, gE, gG, gH, gI, gJ, gK, gL, gM, and gN, as well as 4 non-glycosylated proteins, UL20, UL24, UL34, and UL45. Among these, gC, gD, gH, gL, and gB play crucial roles in viral entry into host cells(Campadelli-Fiume et al. 2000; Gianni et al. 2009a; Di Giovine et al. 2011; Dolan et al. 1998). The virus also expresses several regulatory and structural proteins critical for its replication and immune evasion. HSV-1 selectively develops lifelong latency in the trigeminal ganglia (oral), whereas HSV-2 is evolutionarily Optimized for the sacral ganglia (genital), even when the replication pathway is conserved. Their unique tissue tropism and effectiveness in avoiding local immune responses are determined by surface proteins such as gG and small differences in gB/gD binding.

The initiation of viral entry is mediated by the receptor-binding protein gD, which interacts with three distinct classes of receptors: Nectin-1 (an Ig superfamily protein), the Herpes Virus Entry Mediator (HVEM, a member of tumor necrosis factor superfamily) and 3-O-sulfonated derivatives of heparan sulfate (3-OST HS). The C-terminal region of gD overlaps with the Nectin-1 and HVEM binding sites on gD. When gD interacts with host cell receptors, it undergoes a conformational change, and the C-terminal region, known as the Profusion domain (PFD), interacts with the gH/gL complex (Gianni et al. 2009b). This complex regulates the fusion process by converting gB from a prefusion state to a fusion-activated state (Figure S1).(Cooper and Heldwein 2015). While this entry mechanism represents the front door of infection, current clinical interventions focus almost exclusively on the engine room of the virus. Acyclovir (ACV) and its derivatives remain the cornerstones of antiviral treatment for herpes simplex virus (HSV) infection because they target viral DNA polymerase to inhibit viral DNA synthesis. ACV is a selective deoxyguanosine analogue that is phosphorylated by viral thymidine kinase and subsequent cellular kinases to form Acyclovir triphosphate, which is incorporated into viral DNA, causing premature chain termination and irreversible inhibition of DNA polymerase activity(James and Prichard 2014). Acyclovir’s limited oral bioavailability and increasing incidence of resistance, particularly in immunocompromised patients due to mutations in viral thymidine kinase and DNA polymerase genes, complicate its management despite its efficacy and favourable safety profile (Steingrimsdottir et al. 2000). These difficulties highlight the urgent need for alternative treatments with improved pharmacological characteristics and effectiveness.

Compared with traditional small molecules, a distinct and quickly growing class of medicines known as ‘peptide therapeutics’ offer promising advantages, including higher specificity and fewer side effects (Lau and Dunn 2018). These molecules, which are made up of short chain amino acids (usually 2 to 50 residues), are popular in drug design for their high target specificity and antibody efficacy while maintaining a reduced size that permits tissue penetration and improved manufacturing complexity. Peptides are developed to imitate or oppose natural signalling molecules, in contrast to conventional medication therapy. Peptide-based medicines have advanced to the forefront of treatment for infectious illnesses, cancer, and metabolic disorders because of their favourable safety profiles and minimal systemic toxicity as they are metabolised into natural amino acids(Lamers 2022). However, compared with traditional medications, their use in viral therapies is significant. While current nucleotide analogues primarily function by inhibiting intracellular viral replication, they can only intervene after the virus has already successfully penetrated the host cell. Peptides derived from the human HVEM receptor disrupt the interaction between HSV glycoprotein D (gD) and its receptor, effectively blocking viral entry at an early stage of infection(Antoine et al. 2013). Such approaches address the critical limitation of current drugs that predominantly target viral replication without curing infection, offering the potential for therapies that prevent viral entry, reduce resistance development, and ultimately provide curative outcomes. This research aims to develop a novel host derived peptide therapeutics against the herpes simplex virus by integrating computational design with experimental validation. Candidate peptides were first screened and optimized in silico using Schrödinger suite to predict high potency binders, followed by in vitro antiviral activity confirmation and therapeutic validation.

## Materials

The HVEM-derived peptide NP (KEACGELTGTVCEP) and modified peptide screened after residue scanning F4 (RQACGELTGTVCEP) (derived residues 26–39; purity > 90%) were purchased from GenScript (USA). The Vero cell line (African green monkey kidney cells) was purchased from the National Centre of Cell Sciences (NCCS), Pune. Acyclovir was purchased from Sigma-Aldrich (St. Louis, MO). Thiazolyl blue tetrazolium bromide (MTT), Dulbecco’s modified Eagle’s medium (DMEM) and phosphate-buffered saline (PBS) were obtained from HiMedia Laboratories Pvt. Ltd., Mumbai, India. The HSV-1 virus strain SC-16 was procured from Christian Medical College, Vellore, Tamil Nadu and is maintained in our laboratory at the Department of Pharmaceutical Biotechnology, MCOPS.

All computational analyses were performed using Schrödinger Software Suite (Schrödinger release 2025-1; Schrödinger, LLC; New York, NY).

## Methods

### Selection of amino acids and 3D structural design of the derived peptide

The primary sequence of HVEM, a TNF receptor superfamily member, was retrieved from the UniProt database (accession number: Q92956) (https://www.uniprot.org). On the basis of the established structural data, a 14-mer peptide (NP) corresponding to residues 26–39 was selected for the study. This particular area was shown to play a crucial role in the binding interface, enabling direct contact with the HSV glycoprotein gD. The 3D structure of the peptide was modelled *de novo* using the advanced *de novo* peptide structure prediction server PEP-FOLD-3 on the RPBS portal (https://bioserv.rpbs.univ-paris-diderot.fr/services/PEP-FOLD3/). The derived peptide structures were validated using the PROCHECK server (https://saves.mbi.ucla.edu/) (Laskowski et al. 1993).

### Molecular docking of native peptide

#### Prediction of the physicochemical properties of the peptides

The physicochemical properties of the derived peptide, including molecular weight, theoretical pI, total number of positively and negatively charged residues, and stability index, were calculated using the ExPASy-ProtParam tool (https://web.expasy.org/protparam/) (Gasteiger 2003), whereas PepCalc (https://www.pep-calc.com/) an online peptide calculator was utilised to predict peptide solubility, molecular weight verification and automatic mass spectral peak assignment to confirm peptide purity (Lear and Cobb 2016). The instability index was specifically utilised to estimate the in vivo stability of the sequences, with values less than 40 indicating a stable configuration. This index was calculated as Instability Index = 10/×(ℎ) × ×(+1),

Where L: - Length of sequence.

i: - L-1.

If instability index is less than 40, then the peptide is supposed to be stable.

#### Molecular docking and binding interface analysis of native peptide

The interaction between HSV-1 gD and the HVEM-derived peptide was studied by molecular docking using the Schrödinger Maestro suite. The crystal structure of HSV-1 gD (PDB ID: 1JMA) was retrieved from the Protein Data Bank because of its superior resolution (1.9 Å). The structures were prepared via the Protein Preparation Wizard to optimise hydrogen bonding and assign proper ionisation states. A receptor grid centred on the N-terminal residues identified as critical for HVEM contact, specifically, Gln27, Leu28, Thr29, and Asp30, was generated. The HVEM-derived peptide was docked as a flexible ligand against the rigid gD receptor, and binding interactions for the top-ranked poses, including hydrogen bonds, hydrophobic contacts, and salt bridges, were characterised using the Protein-Ligand Interaction Profiler. The final poses were exported as PDB files for further conformational analysis.

### Molecular dynamics simulations of native peptide

To validate the docking results and assess the complex stability of the peptide and gD complex, molecular dynamics (MD) simulation was performed via Desmond. The complex was prepared under an OPLS3e force field (Kotadiya et al. 2025) and solvated within an orthorhombic box using the TIP3P water model to simulate a physiological aqueous environment. A production run was executed for 100 ns under NPT ensemble conditions, maintaining a constant temperature of 300 K and a pressure of approximately 1.01325 bar. The simulation trajectories were processed and analysed via a simulation interaction diagram (SID) to evaluate the root mean square deviation (RMSD) and root mean square fluctuation (RMSF), and to evaluate protein-peptide intermolecular contacts over time (Kotadiya et al. 2025).

### In Silico residue scanning and lead optimization

To enhance binding affinity and structural stability, the native HVEM-derived peptide was subjected to computational residue scanning using the Protein Engineering module in the Schrödinger Biologics Suite. The energy-minimised structures of the peptide-gD protein complex served as a template for systematic mutagenesis. For each residue position, the protocol was used to calculate changes in binding affinity (ΔΔG _binding_) and structural stability (ΔΔG _stability_) relative to those of the wild-type sequence. Mutations that yielded significantly negative ΔΔG _stability_ while maintaining structural integrity were prioritised. The most promising lead candidate was selected for further validation. This Optimized sequence was then subjected to physicochemical characterisation., such as their molecular weight, pI, and net charge, and stability of peptide which were predicted via the ProtParam algorithm of the ExPASy (https://web.expasy.org/protparam/) web server. PepCalc.com (https://pepcalc.com/) was used to predict the solubility of the peptides, and the 3D structure was predicted via the PepFold 3 web server (Lamiable et al. 2016). The structures were subjected to molecular docking and 100 ns molecular dynamics simulations with the similar parameters as n ative peptide to determine the superior performance of the modified peptide-gD complex.

### In vitro anti-HSV screening of native and modified peptides

#### Cells and reagents

The Vero cell line was cultured in Dulbecco’s modified Eagle’s medium (DMEM) supplemented with 10% fetal bovine serum (FBS) and 1% antibiotic-antimycotic solution. The cultures were incubated at 37 °C and 5% CO_2_. For all the experimental assays, maintenance media with a reduced serum concentration with 1% FBS was used to limit cell proliferation.

#### Solubility of the peptides

Working solutions of the native peptide and its modified derivative were formulated by diluting the stock in maintenance media. For the cytotoxic evaluation, cells were incubated in maintenance medium with 3% heat inactivated FBS. Conversely for antiviral activity assay maintenance medium containing 2% FBS was utilised. Throughout the dilution process, careful monitoring was maintained to ensure complete solubility and to avoid peptide precipitation, ensuring consistent delivery of the intended concentrations.

#### Cytotoxicity assay

The cytotoxicity of the native peptide and modified peptide was assessed via a 3-(4,5-dimethylthiazol-2-yl)−2,5-diphenyltetrazolium bromide (MTT) assay. Cells were seeded in 96-well plates at a density of 1 × 10^4^ per well and incubated for 24 h at 37 °C in a 5% CO2 atmosphere to allow for adherence. Peptide stock solutions were prepared and serially diluted in maintenance media to achieve final concentrations of 15.62, 31.25, 62.5, 125, and 250 µg/mL. Each concentration was added to the wells in triplicate. Following a 72-hour incubation period with daily microscopic monitoring, 50 µL MTT reagent (5 mg/mL) was added to each well. After a further 4hr incubation, the supernatant was removed and replaced with 100 µL of DMSO to solubilise the resulting formazan crystals (Yuen et al. 2023). The absorbance was measured at 570 nm via an ELISA plate reader. The percentage of cytotoxicity was calculated via the following formula:

$$\% {\mkern 1mu} Cytotoxicity{\mkern 1mu} = {\mkern 1mu} \frac{{Absorbance{\mkern 1mu} \,of{\mkern 1mu} control{\mkern 1mu} - {\mkern 1mu} Absorbance{\mkern 1mu} \,of{\mkern 1mu} drug}}{{Absorbance{\mkern 1mu} \,of{\mkern 1mu} control}} \times 100$$

#### Cytopathic effect (CPE) inhibition antiviral assay

The antiviral activity of the peptides was assessed by evaluating their ability to inhibit the HSV-1-induced cytopathic effect in Vero cells. The peptides were dissolved in sterile distilled water to prepare a stock solution of 5 mg/1mL. Vero cells were seeded into 96-well microtiter plates at a density of 1.5 × 10^4 cells/well and incubated for 24 h at 37 °C in a humidified atmosphere with 5% CO_2_ to allow cell attachment and monolayer formation. Vero cells were incubated with 100 µl of the virus suspension containing 10 TCID50 (50% tissue culture infective dose-Figure S2) per well in a 96-well plate. Following a 2-hour adsorption period at 37 °C, the viral inoculum was removed and replaced with 100 µL of peptide solution at four distinct concentrations. For initial antiviral screening of the native peptide was evaluated in a single screening cycle, whereas the optimized variant was tested in four independent experimental cycles. All treatment concentrations were tested in quadruplicate, with Acyclovir ($\:25\:\mu\:\mathrm{g/mL}$) serving as the positive control. The cell controls consisted of uninfected cells in maintenance media (DMEM with 2% FBS), while the virus controls consisted of infected cells without peptide treatment. The plates were incubated at 37 °C under 5% CO2 for five days. To determine the degree of viral inhibition, the CPE was monitored microscopically(Cotarelo et al. 1999; Gong 2013; Rahmasari et al. 2020). At the end of the incubation period, 100 µL of MTT reagent (5 mg/mL in PBS) was added to each well and incubated for 4 h. The resulting formazan crystals were dissolved in 100 µL of DMSO, incubated for 20 min at 37 °C, and quantified at 570 nm using an ELISA plate reader.(Cotarelo et al. 1999; Rahangdale et al. 2025a).

The percentage of CPE inhibition was calculated via the following formula:

$$\begin{array}{*{20}{l}}{\% CPE{\mkern 1mu} inhibtion{\mkern 1mu} = {\mkern 1mu} \left( {Abs.{\mkern 1mu} of{\mkern 1mu} the{\mkern 1mu} peptide{\mkern 1mu} treated{\mkern 1mu} \inf ected{\mkern 1mu} cell} \right) - \left( {Abs.{\mkern 1mu} {\mkern 1mu} of{\mkern 1mu} the{\mkern 1mu} viral{\mkern 1mu} control} \right){\mkern 1mu} {\mkern 1mu} }\\{\quad \quad \quad \quad \quad \quad \quad \quad /\left( {Abs.{\mkern 1mu} of{\mkern 1mu} the{\mkern 1mu} cell{\mkern 1mu} control} \right) - \left( {Abs.{\mkern 1mu} of{\mkern 1mu} the{\mkern 1mu} viral{\mkern 1mu} control} \right) \times 100}\end{array}$$

## Results

### Selection of amino acids and 3D structural design of the native peptide

The interaction between the HVEM and gD is critical for HSV entry into host cells, as it triggers the essential conformational changes in the viral envelope required for membrane fusion. This binding interface primarily involves the gD N-terminal region and the HVEM contact region. Specifically, key residues of gD M11, N15, L25, Q27, L28, T29, D30, and R35 form robust contacts with HVEM residues D7, E8, C16, T35, P39, R75, and S74-T76 of HVEM, with particularly high affinity noted for E8 and T35(Yoon et al. 2003). This receptor-ligand engagement initiates a signalling cascade that activates the viral fusion machinery, which is composed of gB and the gH/gL complex (Cairns et al. 2020). Consequently, we targeted this early-stage interaction by deriving a competitive inhibitory peptide from the HVEM binding domain. By mimicking the natural receptor interface, this peptide aims to occupy the gD binding site, thereby neutralising the virus before it can initiate the fusion process and establish infection.

###  Molecular docking of native peptide

#### Prediction of the physicochemical properties of the peptides

**Fig. 1:**
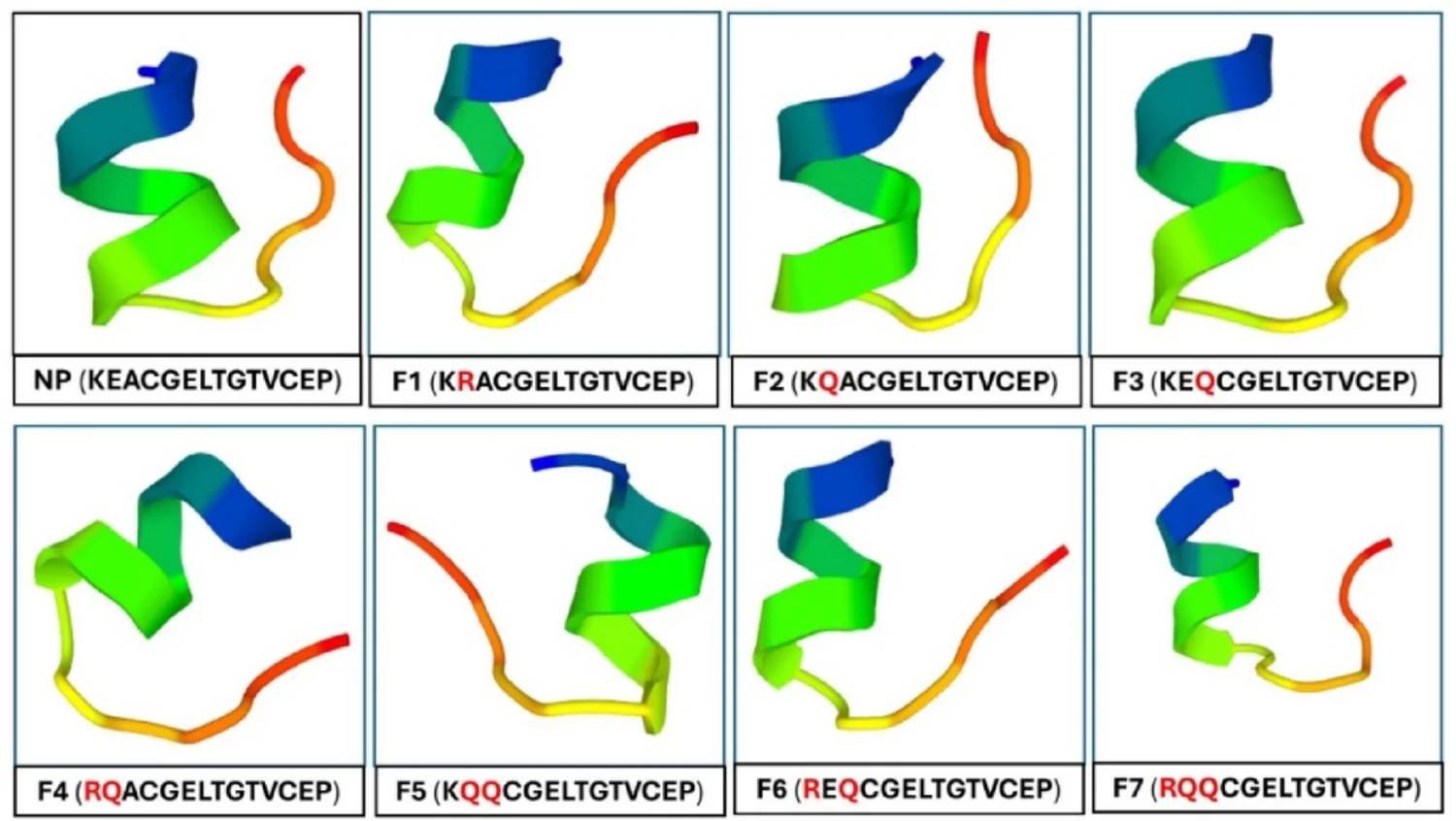
Ribbon models of the 3D structure of the Native Peptide (NP) and the series of Modified Peptides (F1-F7) designed through the PEPFOLD3 tool

The 3D structure of the HVEM-derived peptide was modelled *de novo* via the PepFold 3 web server (Fig. 1). The model shows a clear α-helical (α-helix) conformation in the central region (the coiled ribbon), flanked by more flexible random-coil regions at the N- and C-termini (the thin, wire-like ends). The physicochemical properties of the peptide sequence KEACGELTGTVCEP were calculated revealing a 14-residue molecule with a molecular weight of 1436.61 Da.

With a theoretical isoelectric point (pI) of 4.25 and a net charge of −2, the peptide was found to be acidic, stable and having a negative charge at physiological pH. Its solubility index of 36.91 suggests moderate hydrophilicity, and computational analysis classified it as a non-toxin, indicating a favourable safety profile for further biological testing (Table 1).

**Table 1:** Physicochemical properties and protein-peptide docking and interaction analysis of native (NP) and the modified peptides (F1-F7)

Code	Sequence	No. of residues	Molecular weight	Theoretical PI	Net charge	Docking Score	Binding free energy (MM/GBSA) (kcal/mol)	Interactive residues at HSV-gD
NP	KEACGELTGTVCEP	14	1437	4.25	−2	−8.626	--	Asp (30), Gln (27), Leu (28), Asp (30), Arg (36)
F1	REACGELTGTVCEP	14	1464	3.85	−2	−8.134	−52.29	Asp (26), Gln (27), Arg (36)
F2	KQACGELTGTVCEP	14	1435	4.15	−1	−7.407	−30.42	Gln (27), Thr (29), Asp (30), Arg (36)
F3	KEQCGELTGTVCEP	14	1435	4.15	−1	−7.985	−32.67	Asp (30), Gln (33), Arg (36)
F4	RQACGELTGTVCEP	14	1463	4.15	−1	−8.710	−77.33	Arg (18), Gln (27), Thr (29)
F5	KQQCGELTGTVCEP	14	1492	4.15	−1	−7.883	−12.19	Gln (27), Thr (29), Asp (30), Val (34), Arg (36)
F6	REQCGELTGTVCEP	14	1521	3.85	−2	−8.543	−56.47	Gln (27), Asp (30), Gly (33), Arg (35), Arg (36)
F7	RQQCGELTGTVCEP	14	1520	4.15	−1	−6.997	−28.19	Gln (27), Thr (29), Asp (30)

#### Molecular docking analysis of native peptides

**Fig. 2:**
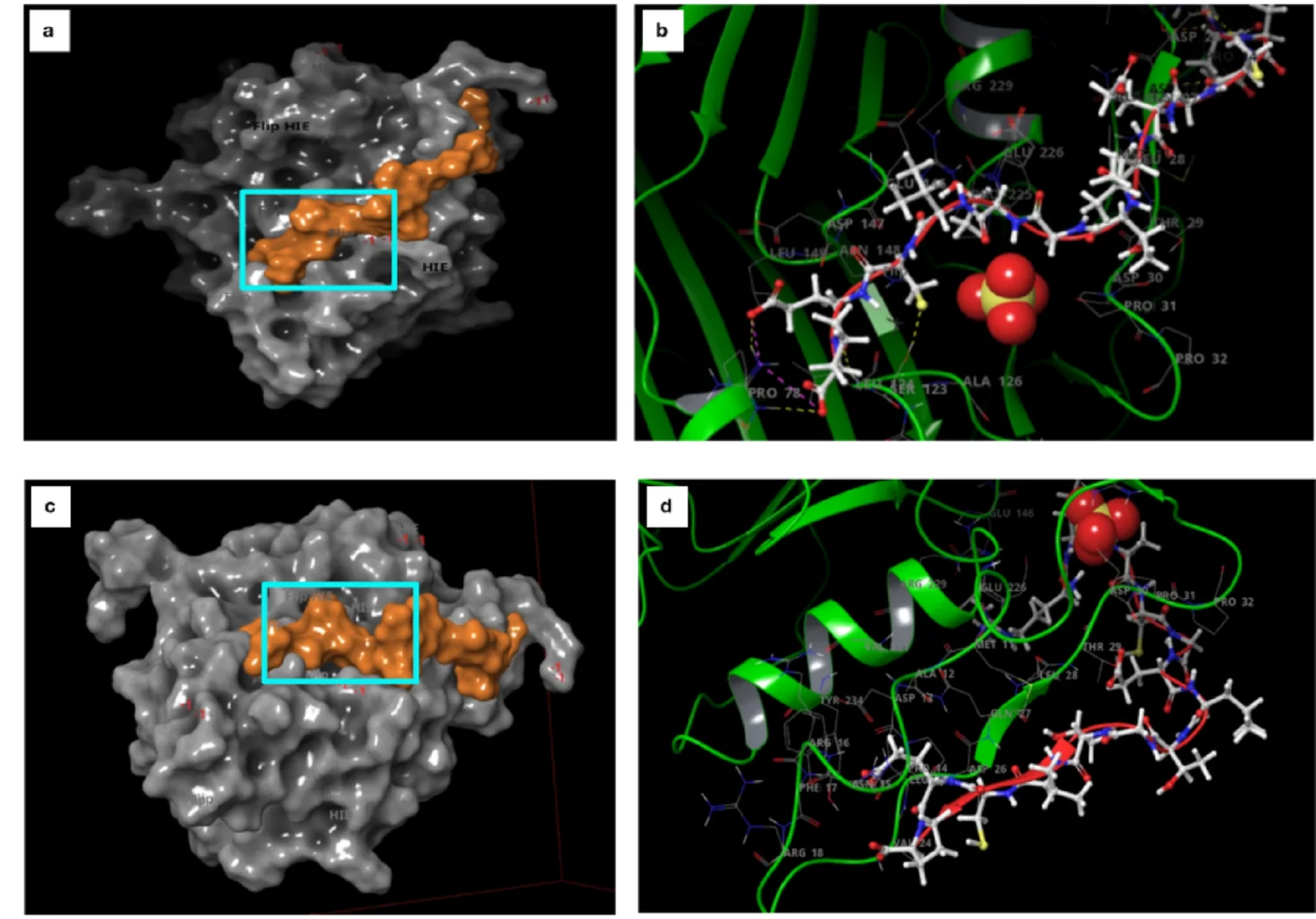
Molecular docking and surface complementarity of HVEM-derived peptides with HSV-1 glycoprotein D (gD). **a** Surface representation of the gD protein (grey) complexed with the native peptide NP, (orange), highlighting the spatial complementarity at the binding interface. **b** 3D docking pose of NP (ball and stick) within the gD binding pocket (green ribbon). The peptide backbone is shown in red, with side chains colored by atom type (C: white, N: blue, O: red, S: yellow). **c** Surface representation of the gD protein (grey) in complex with the modified peptide F4, (orange), demonstrating maintained or enhanced interface fit. **d** 3D docking pose of F4 (ball and stick) complexed with gD (green ribbon), illustrating the structural orientation of the modified residues within the binding site

To evaluate the inhibitory potential of both peptides, molecular docking was performed against the HSV-1 gD receptor (PDB: 1JMA). The resulting binding pose revealed a high-affinity interface stabilised by specific atomic contacts. The interaction between HSV-1 glycoprotein D (gD) and the peptide involves several specific contacts that aid in viral entry. (Fig. 2a).

To evaluate the binding efficacy of HSV-1 glycoprotein D (gD) and the native and newly derived peptide sequences, site-specific molecular docking was conducted using the Schrödinger Maestro Suite. The objective of this study was to determine the spatial orientation and binding affinity of the peptide within the HSV-1 gD binding pocket. Comparative analysis of native and modified peptide docking poses focused on assessing the maintenance of critical contacts with the reported gD hotspots, which are essential for viral infection. The native peptide demonstrated a binding affinity against HSV-gD, yielding a docking score of −8.626 kcal/mol. This interaction is primarily stabilized by key contacts with specific amino acid residues on the HSV-gD target, including Asp30, Gln27, Leu28, and Arg36.The natural peptide was found to have a unique binding footprint that simultaneously binds to Asp26 and Leu28, even though it also targets the Gln27-Asp26 region.

### Molecular dynamics simulations of native peptide

**Fig. 3:**
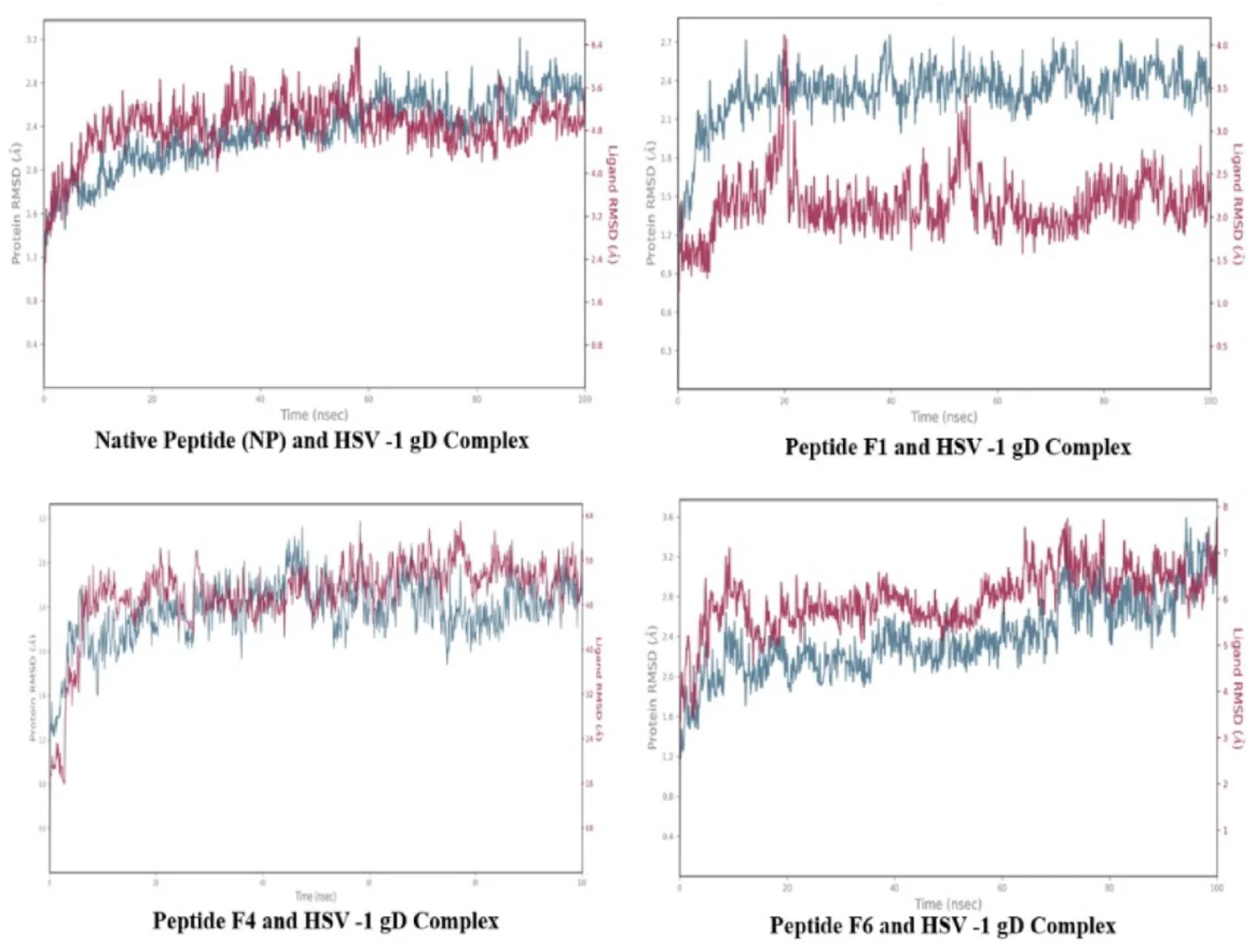
RMSD plot of the Native peptide (NP) and Modified Peptides F1, F4 & F6 with the HSV-1 gD protein at 100 ns showing the stability and binding dynamics

The RMSD plot shown in Fig. 3 provides an essential assessment of the ability and behavior of the HSV-1 gD-HVEM-derived peptide complex over a period of 100 nanoseconds (ns). On the x-axis, the plot depicts simulation time (in ns), whereas the y-axis to the left shows the protein RMSD, which is the average deviation of the protein backbone atoms, and the y-axis to the right displays the ligand RMSD values, indicating the peptide’s movement relative to the protein structure. A consistently low RMSD, with fluctuations remaining below 3Å for both the protein and the ligand throughout the trajectory, is indicative of a structurally stable, well-bound complex. Initially, docking predicted that the peptide would form a single hydrogen bond with the gD residue at positions 26 and 27, a critical part of the gD-HVEM antiparallel β-sheet, which was observed to form stable hydrogen bonds with the peptide. These dynamic interactions further confirmed that the designed peptide can successfully interrupt the critical gD-HVEM interface, according to the in silico predictions. Overall, the RMSD analysis provides compelling evidence that both the protein and peptide components of the complex remained conformationally stable, supporting the efficacy and reliability of the peptide as a potential antiviral agent for disrupting HSV entry. In simulations of the native peptide, the dual interaction with Asp26 and Leu28 was found to be structurally crowded, creating a persistent steric shield over Gln27. This hindered the ability of the peptide to achieve the deeper pocket penetration required for maximum inhibitory effects.

###  In silico residue scanning and lead optimization

Computational Residue scanning was conducted to systematically evaluate the energetic impact of point mutations at each position of the native peptide sequence. This approach enabled the identification of specific amino acid substitutions that influence overall binding affinity (ΔΔG_bind_) and complex stability (ΔΔG_stability_) upon binding to the target protein. Rather than relying solely on spatial interaction patterns, our selection criteria prioritised mutations that exhibited favourable (negative) ΔΔG_bind_ values for both affinity and structural integrity. Residues demonstrating significant improvements in these thermodynamic parameters were identified as mutational hotspots, providing a rational foundation for the development of Optimized variants.

Novel peptide variants were generated by introducing targeted single or double amino acid mutations at the most promising positions. Table 2 summarises the highest-performing substitutions at each residue position. For the sake of brevity and clarity, only mutations that yielded positive improvements in both ΔΔG_bind_ and ΔΔG_stability_ are represented. This data-driven strategy enabled the transition from the native sequence to a refined lead candidate, which was subsequently subjected to comparative molecular docking and future structural modelling to validate its enhanced performance.

**Table 2:** Residue scanning mutation effect on affinity and stability

Mutation	Delta Affinity	Delta Stability
C:11 (VAL-> ARG)	−0.91585	−10.4647
C:10 (THR-> GLN)	−7.0444	−1.73182
C:10 (THR-> MET)	−15.1341	−19.5349
C:8 (THR-> PHE)	−12.3262	−3.76374
C:8 (THR-> TYR)	−11.6577	−3.42656
C:7 (LEU-> ARG)	−5.14269	−5.36528
C:6 (GLU-> PHE)	−2.95286	−3.57179
C:6 (CLU-> VAL)	−2.50826	−3.17516
C:3 (ALA-> ARG)	−0.56974	−6.27334
C:3 (ALA-> ASN)	−0.6534	−5.88873
C:2 (GLU-> GLN)	−0.15601	−6.47752
C2: (GLU-> TRP)	−0.57491	−1.30336
C1: (LYS-> ARG)	−1.09707	−18.3704
C1: (LYS-> MET)	−0.77908	−18.2996

#### Rational design and structural modelling of optimized variants

Based on the thermodynamic profiles obtained from residue scanning, seven novel peptide variants (F1-F7) were rationally engineered to enhance the binding efficacy and stability of the lead sequence, NP (Table 1). The designed strategy focused on incorporating single and double amino acid substitutions at non-critical positions to optimise the global free energy of the complex without disrupting essential pharmacophores. Table 1 lists the physicochemical properties of seven 14-residue peptide variants, designated F1-F7, which are derivatives of a common core sequence. Each variant maintains a consistently high safety profile, with all seven being classified as nontoxic and exhibiting good solubility, making them suitable candidates for cell-based antiviral assays. The molecular weights of these peptides are closely clustered between 1435 and 1521 Da, with slight variations resulting from specific N-terminal amino acid substitutions. Structurally, these peptides are acidic, as evidenced by their low theoretical isoelectric points (pIs) ranging from 3.85 to 4.15. Consequently, they carry a net negative charge of either − 1 or −2 at physiological pH, a characteristic that significantly influences their electrostatic interactions with both viral glycoproteins and the Vero cell surface during CPE inhibition assays. The 3D structural conformations were subsequently modelled *de novo* via the PEP-FOLD3 server to verify that the introduced mutations did not induce unfavourable folding patterns (Fig. 2). The water solubility and toxicity profiles of both the native and modified peptides were evaluated using ToxinPred. This in silico analysis predicted that both sequences possess high water solubility and are non-toxic.

##### Structural constraints and mutation strategy

Particular emphasis was placed on residues at positions 4, 12 and 13, as these residues are known to be essential for interaction with the HSV gD receptor. These critical residues were either retained or conservatively substituted to maintain target engagement. Additionally, residue 14 was recognised as vital for maintaining structural integrity. Conversely, residue 14 was identified as a structural anchor required to maintain the peptide’s bioactive conformation. While cyclic residues such as proline are frequently used to stabilise secondary structures, mutation at position 14 was explicitly avoided. Preliminary structural evaluations indicated that the rigidity imposed by a proline substitution at the C-terminus would place excessive conformational constraints, potentially hindering the molecular flexibility required for optimal docking within the gD binding pocket.

#### Molecular docking analysis of modified peptides

To evaluate the binding efficacy of HSV-1 glycoprotein D (gD) and the mutated peptide sequences, site-specific molecular docking was conducted using the Schrödinger Maestro Suite against gD receptor (Fig. 2a).

The modified peptide demonstrated a highly specific docking configuration, exhibiting superior spatial complementarity within the gD receptor pocket. A key feature of this interaction is the dense network of hydrogen bonds and polar contacts focused on the Gln27 residue of the gD protein. In particular, many hydrogen bonds were observed between Gln27 and the peptide backbone, indicating that a high-affinity anchor point stabilised the complex. Additionally, the modified peptide efficiently seals the binding groove by interacting with Leu25, Asp26, and Thr29 through localised hydrophobic and polar interactions (Fig. 2b) (Table 2).

Compared with the modified form, this simultaneous engagement may restrict the depth of the interaction by producing a steric shielding effect over Gln27. The ability of the redesigned peptide to adopt a more focused binding position aligns with its expected physical features, notably the strong beta-strand propensity (peaking at ~ 6.7) in its core L-T-G-T-V-C motif. A more accurate fit into the HVEM binding pocket on gD is probably enabled by this structural stiffness, which mimics and blocks the natural receptor-ligand interaction. following initial virtual screening, a multi-parametric filtering strategy was employed to prioritize candidates for dynamic validation. Based on a comparative evaluation of binding poses, structural complementarities at the interface, docking scores, and calculated binding free energies ($\:{\Delta\:}{G}_{\mathrm{bind}}$ via MM-GBSA), the top three mutants, F1, F4, and F6 were selected. To comprehensively evaluate their structural stability, conformational adaptations, and sustained target engagement over time, these three lead variants were subjected to detailed MD simulations.

#### Molecular dynamics simulations of modified peptide

The molecular dynamics simulations for the modified peptides were executed using the identical system setup, parameterizations, and production configurations applied to the native peptide. From the shortlisted peptides, F1, F4, and F6 (Fig. 3), the variant F4 demonstrated superior stability in the MD simulation, as reflected by a consistently low and stable RMSD profile for both the protein and ligand throughout the simulation period. These findings indicate that the peptide maintained a stable binding pose with minimal deviation and that the overall complex reached equilibrium early and remained stable. In addition to the RMSD, visual inspection and interaction analysis further indicated that this peptide-maintained interactions with key binding residues of gD over time. The retention of hydrogen bonds and hydrophobic interactions observed during simulation support the structural compatibility and binding resilience of the peptide, indicating that it is a better candidate for further development. Hence, this final peptide (F4) was selected as the Optimized lead variant because of its favourable binding profile, enhanced structural stability, and potential to outperform the native peptide in terms of therapeutic effectiveness against HSV-1.

The MD trajectories provided a dynamic explanation for the superior performance of the modified peptide. Conversely, to the native peptide, the modified peptide (Figure S3 & S4) displayed an Optimized binding footprint that avoided this steric clash, allowing Gln27 to participate in a more robust and direct hydrogen-bonding network. The stability of the modified complex is largely attributed to the central L-T-G-T-V-C motif, which has a high β-strand propensity, peaking at 6.7. During the trajectory, this region prevents the dissociation observed in less rigid sequences. This rigidity ensures that the critical inhibitory contacts with Gln27 remain consistent throughout the simulation.

### In vitro evaluation of the effects of HVEM-derived peptides against HSV-1

####  Solubility of peptide

The aqueous solubility of both NP and F4 was evaluated to confirm their suitability for in vitro testing. Both peptides were found to be freely soluble in water, a property attributed to their anionic nature, which promotes favourable electrostatic interactions with polar water molecules and ensures efficient dissolution. High aqueous solubility is a critical requirement for bioactive peptides, as it simplifies formulation, enhances ease of handling, and ensures compatibility with biological assays. For the experimental treatments, a twofold serial dilution of both peptides was prepared, resulting in final working concentrations of 250 µg/mL, 125 µg/mL, 62.5 µg/mL, 31.25 µg/mL, and 15.62 µg/mL.

#### Cytotoxicity assay

The safety profile of the peptide was validated using an MTT assay in Vero cell monolayers. Neither the native nor modified variants induced significant cytotoxicity even at the maximum concentration of 250 µg/ml. The cell viability remained consistent (Figure S5), confirming that the peptides are nontoxic to the host cells. This high degree of biocompatibility establishes a broad therapeutic window for the subsequent evaluation of their antiviral efficacy(Ghandehari et al. 2015). Consequently, the CC_50_ for both peptides was determined to be ≥ 250 µg/ml. This high degree of biocompatibility suggests a highly favorable safety profile, establishing a broad therapeutic window for subsequent evaluations.

#### Cytopathic effect (CPE) inhibition antiviral assay

**Fig. 4:**
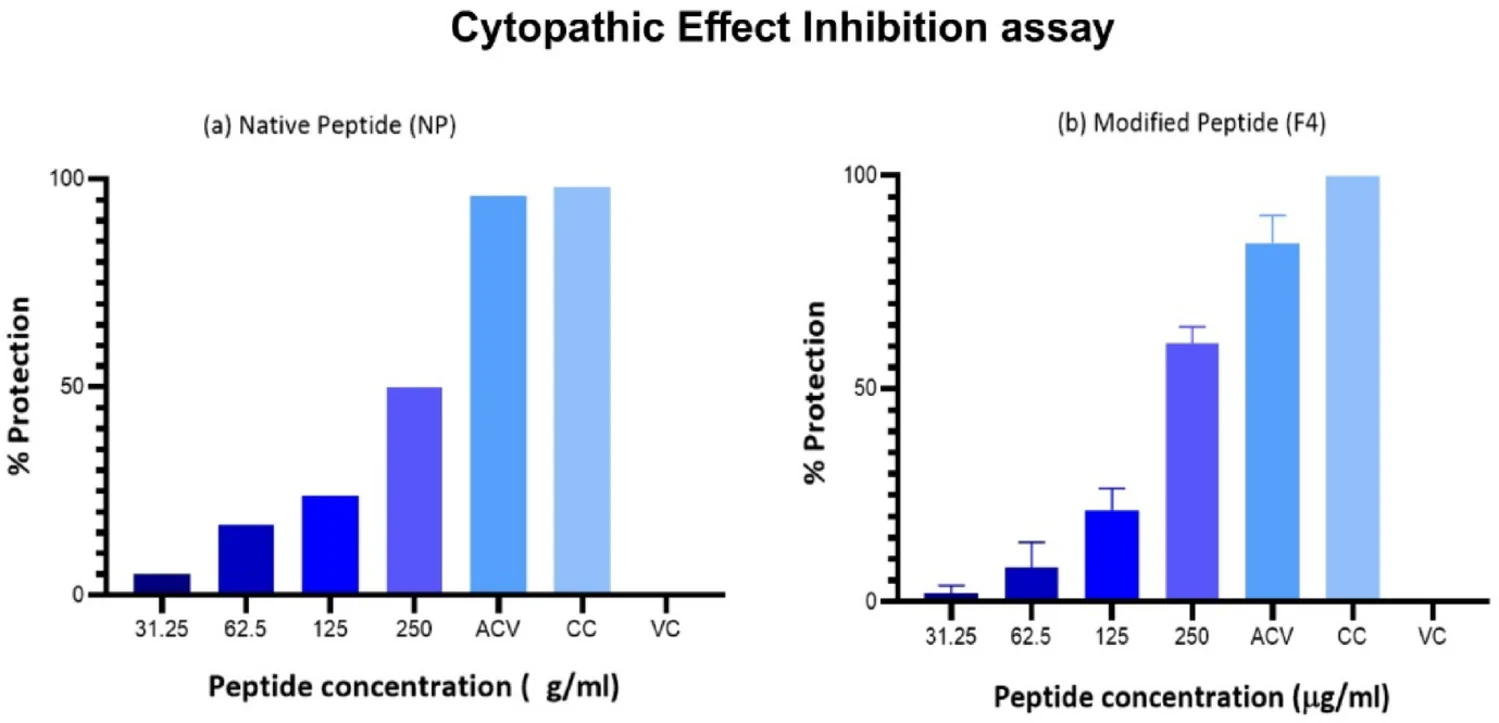
Antiviral activity of the **a** Native Peptide (NP) and **b** Modified Peptide (F4) studied against HSV-1 infection through a cytopathic inhibition assay. The graph illustrates the dose-dependent effects of NP and F4 on Vero cell protection. The Cell Control (CC) maintained 98% and 99% viability, while the Virus Control (VC) exhibited 0% protection. A periodic increase in the protection percentage was observed with increasing peptide concentration. Acyclovir (25 µg/mL), used as a positive control, showed the highest antiviral activity, with 96% in Native peptide sample testing and 84% protection for modified peptide

The antiviral efficacy of the native peptide NP and its Optimized derivative, F4, was evaluated using a CPE inhibition assay against HSV-1 infection in Vero cell lines. Both peptides clearly inhibited viral replication in a dose-dependent manner. At the maximum tested concentration of 250 µg/mL, NP inhibited CPE by approximately 50% In comparison, the reference antiviral drug Acyclovir (ACV), tested at a significantly lower concentration (25 µg/mL), provided superior protection, with 96%. In contrast, the rationally designed F4 variant exhibited enhanced antiviral protection, reaching 60.58% inhibition at the same concentration. (Fig. 4). The efficacy of F4 followed a distinct dose-response relationship, decreasing to 21.56% inhibition when the concentration was reduced to 125 µg/ml. when the ACV protection was 84%. Although the peptide exhibited lower potency than the clinical standard ACV did, both demonstrated significant viral neutralisation at higher doses(Rahangdale et al. 2025b). The experimental results correlate strongly with the in silico docking and molecular dynamics data, which predicted that the specific modification in F4 would enhance the binding stability with HSV-1 gD. The superior performance of the modified peptide over the native NP sequence validates our rational design approach. (Raw Data: Table S3)

## Discussion

Traditional anti-HSV research targeting the entry phase has frequently focused on blocking viral envelope glycoproteins with neutralising monoclonal antibodies or small-molecule inhibitors; however, these approaches often face challenges due to high production costs or suboptimal target specificity. In contrast, peptide-based strategies offer a highly tunable, biocompatible alternative. While previous studies have successfully developed peptides targeting glycoprotein B (gB) or glycoprotein H/L (gH/gL), strategies that explicitly focus on the gD interface, utilising host-receptor derivation, are particularly powerful. This research demonstrated that disrupting the protein-protein interaction between viral glycoprotein D (gD) and the host HVEM receptor is a robust strategy for developing novel antiviral therapeutics against HSV-1. Initial evaluation of a native HVEM-derived peptide (NP) showed a favourable safety profile and moderate antiviral efficacy, achieving up to 50% protection in vitro. Through a systemic, data-driven Optimization approach integrating in silico residue scanning with experimental validation, we engineered several peptide variants with enhanced antiviral activity. Advances in silico analysis, including molecular docking, molecular dynamics simulation, and MM-GBSA calculations, have demonstrated the enhanced binding affinity and greater stability of the Optimized peptide. The lead candidate F4 exhibited modest enhancement in performance characterised by enhanced binding affinity, structural robustness and dose-dependent protection. Molecular docking against the 1JMA viral template and MM-GBSA calculations indicated superior thermodynamic stability compared with the native sequence. Molecular dynamics simulations showed that the Optimized substitutions at noncritical residues (positions 4, 12, and 13) improved the ability of the peptide to maintain the bioactive hairpin conformation required for target engagement. In vitro assays demonstrated the therapeutic potential of F4, which achieved a maximum protection of 60.58% against HSV-1. While the antiviral efficacy of the peptides was lower than that of the gold standard Acyclovir (84.16%), the distinct entry-inhibition mechanism of F4 offers a slight advantage as a complementary or alternative therapy, particularly in the management of Acyclovir-resistant strains. It is important to note that while Acyclovir is used as a standard reference control in our assays, it operates as a nucleoside analogue inhibiting viral DNA replication, whereas peptide F4 is designed to block entry. Because these agents target distinct stages of the HSV-1 life cycle, direct comparisons of their efficacy profiles should be interpreted with caution. While the current efficacy of F4 mutant demonstrates modest improvement over that of native sequence with %protection increasing from 50% to 60.58%, this steady increment underscores the validity of targeted residue substitution. However, it is important to note that this represents an Optimized starting point rather than a final ceiling for potency. To fully elucidate the precise mechanism of action of the designed peptide, further mechanistic investigations including plaque reduction assay, time of addition pre and post treatment assay has to be done. Even though the current activity range is slightly below that of conventional nucleoside analogues, the modular nature of this peptide allows for further chemical modifications, such as peptidomimetics, backbone cyclisation, or the incorporation of nonnatural amino acids, which are anticipated to further increase proteolytic stability and binding kinetics. Consequently, the iterative refinement demonstrated here suggests that this scaffold possesses the structural plasticity required for future development into a high-potency clinical candidate capable of surpassing current therapeutic limitations.

Furthermore, the high solubility and lack of cytotoxicity observed at concentrations up to 250 µg/mL underscore the clinical viability of this scaffold. Collectively, these findings provide a rational foundation for the continued development of peptide-based antiviral solutions, highlighting the importance of combining computational modelling with laboratory assays to address the global challenge of increasing drug resistance.

### Future aspects

To build upon these promising initial findings, the next phase of our research is positioned to expand this work through several key developmental milestones. While the molecular docking and molecular dynamic simulation in this research support the hypothesis that the optimized HVEM derived peptides target HSV-1 gD, the in vitro data demonstrates broad antiviral activity than the definitive entry inhibition. Because the standard CPE inhibition assay captures an aggregate antiviral effect, advanced mechanistic experiments such as time of addition, viral adsorption and penetration assays are required to definitively isolate the exact stage of viral life cycle being blocked. Moving forward, executing these targeted mechanistic assays and exploring potential synergy between these two distinct modes of action will be prioritized to transition these rationally designed peptides into fully validated preclinical leads. We plan to evaluate the peptide’s efficacy in additional HSV-susceptible human cell lines, such as HaCaT or HEp-2, to confirm its therapeutic breadth. Finally, transitioning these candidates into in vivo models will allow us to evaluate their systemic safety, tolerability, and pharmacokinetic profiles, paving the way for advanced preclinical development.

## Supplementary Information

Below is the link to the electronic supplementary material.

Supplementary Material 1

## Data Availability

All data supporting the findings of this study are available within the paper and its Supplementary Information.
