# Supplementary material for: Design, development and enhanced efficacy of host-derived antiviral peptides to prevent herpes simplex virus type 1 infection
Source: Amino Acids. 2026 Aug 13;58:64. doi: 10.1007/s00726-026-03552-y (PMC13759857; doi:10.1007/s00726-026-03552-y)
Supplement: Supplementary file 1 [file 726_2026_3552_MOESM1_ESM.docx]

**SUPPLEMENTARY DATA**

Figure. 1. Depicts the structure and complex life cycle of Herpes Simplex Virus. The multistage process begins with the virus binding to specific receptors on the host cell membrane, such as Nectin-2 or HVEM, followed by fusion with the cell membrane to enter the cytoplasm. After successful entry into the cell, the viral capsid travels to the nucleus for capsid docking, where it uncoats its genome and releases its DNA into the nucleoplasm. The viral DNA then undergoes circularisation and is transcribed, producing α, β, and γ transcripts that are transported to the cytoplasm for translation into viral proteins. These proteins return to the nucleus for capsid assembly, packaging the newly replicated viral genomes into protein shells. The final stages involve the capsids budding through the nuclear envelope and moving through the Endoplasmic Reticulum (ER) and the Golgi apparatus for maturation, ultimately resulting in viral egress, during which new infectious virions are released from the cell membrane to infect neighbouring cells. The given image has been created by using BioRender (<https://app.biorender.com/>) software with highest resolution of 600PSI.

1. **Prediction of proteolytic cleavage sites**

PROCLEAVE_SEQ, a bioinformatics tool (<http://procleave.erc.monash.edu/>) developed to predict protease-specific substrate cleavage sites via a combination of sequence and 3D structural information, was used to predict proteolytic cleavage sites. Nine proteolytic enzymes from the blood were identified, 2 of which were found in the database of this tool. Utilising the most recent version of the MEROPS database ver. 12.5. Procleave performs a BLAST search to map the substrate sequence to the PDB structure 35 and creates a comprehensive 3D structural substrate dataset(Li et al., 2020).

**Result:** The scores for the proteolytic cleavage sites mentioned below are probability scores that are between 0 and 1. If the score is near 0, then the chances of cleavage are low, and if the score is near 1, then the chances of cleavage are high. In both tables mentioned below, the scores are near 0, so the chances of peptide cleavage are very low(Supplement).

**Table S1: Thrombin cleavage site on the HVEM-derived peptide predicted by the ROCLEAVE_SEQ tool**

| Rank | Position | P4-P4' Site | N-fragment (kDa) | C-fragment (kDa) | Score | Family |
| --- | --- | --- | --- | --- | --- | --- |
| 1 | 9 | GELT↓GTVC | 0.94 | 0.70 | 0.139 | S01.217 |
| 2 | 6 | EACG↓ELTG | 0.60 | 1.04 | 0.134 | S01.217 |
| 3 | 5 | KEAC↓GELT | 0.43 | 1.21 | 0.129 | S01.217 |
| 4 | 7 | ACGE↓LTGT | 0.73 | 0.91 | 0.116 | S01.217 |
| 5 | 8 | CGEL↓TGTV | 0.84 | 0.80 | 0.099 | S01.217 |
| 6 | 11 | LTGT↓VCEP | 1.22 | 0.42 | 0.098 | S01.217 |
| 7 | 10 | ELTG↓TVCE | 1.12 | 0.52 | 0.073 | S01.217 |

**Table S2: Plasmin cleavage sites on HVEM-derived peptides predicted by the PROCLEAVE_SEQ tool**

| Rank | Position | P4-P4' Site | N-fragment (kDa) | C-fragment (kDa) | Score | Family |
| --- | --- | --- | --- | --- | --- | --- |
| 1 | 9 | GELT↓GTVC | 0.94 | 0.70 | 0.169 | S01.233 |
| 2 | 7 | ACGE↓LTGT | 0.73 | 0.91 | 0.147 | S01.233 |
| 3 | 10 | ELTG↓TVCE | 1.12 | 0.52 | 0.143 | S01.233 |
| 4 | 5 | KEAC↓GELT | 0.43 | 1.21 | 0.139 | S01.233 |
| 5 | 11 | LTGT↓VCEP | 1.22 | 0.42 | 0.116 | S01.233 |
| 6 | 6 | EACG↓ELTG | 0.60 | 1.04 | 0.106 | S01.233 |
| 7 | 8 | CGEL↓TGTV | 0.84 | 0.80 | 0.080 | S01.233 |

2. **Virus titration to determine the tissue culture infectious dose 50% (TCID50)**

**Virus passaging and titration:** The propagation of HSV-1 was carried out in Vero cell cultures at greater than 90% confluency. After the cells were washed twice with PBS, 200 μL of thawed virus inoculum was added, and the culture was incubated at 37°C for 2 hours with gentle intermittent shaking. Following adsorption, maintenance medium (DMEM with 2% FBS) was added, and the cells were incubated until cytopathic effects, characterised by cell rounding and detachment, were observed. The infected cultures were subjected to three freeze‒thaw cycles, followed by centrifugation at 6000 rpm for 4 minutes. The resulting supernatant was then stored at −80°C as the viral stock. This process was repeated 3 times. For titration, the virus stock was serially diluted by tenfold from 10^-1^ to 10^-8^ and inoculated into Vero cells using 8 replicates per dilution in a 96-well plate. After 5 days of incubation, the cytopathic effects were scored, and the TCID_50_ values were calculated via standard methods

**Result:** The propagation of HSV-1 was carried out in Vero cell cultures at greater than 90% confluency. After the cells were washed twice with PBS, 200 μL of thawed virus suspension was added, and the mixture was incubated at 37°C for 2 hours with gentle shaking. Following adsorption, maintenance medium (DMEM with 2% FBS) was added, and the cells were incubated until cytopathic effects, characterised by cell rounding and detachment, were observed. The infected cultures were subjected to three freeze‒thaw cycles, followed by centrifugation at 6000 rpm for 4 minutes. The resulting supernatant was then stored at −80°C as the viral stock. This passage was repeated 3 times. For titration, the virus was serially diluted tenfold and inoculated into Vero cells in a 96-well plate. After 5 days of incubation, the cytopathic effects were scored, and the TCID_50_ values were calculated via standard methods.

**Figure S2: Tissue culture infectious dose 50% calculated by Reed & Muench calculator.**

Figure_S3: 2D ligand-receptor interaction map showing the binding of the peptide sequence KEACGELTGTVCEP (NP). Interactions include hydrogen bonding (pink arrows), salt bridges with charged residues (orange/purple), and water-mediated bridges (grey circles). The high density of interactions in the central region correlates with the predicted structural motif.

**Peptide-HSV-1 gD Complex.**

Figure S4: 2D ligand-receptor interaction map showing the binding of the Modified peptide sequence (F4). Interactions include hydrogen bonding (pink arrows), salt bridges with charged residues (orange/purple), and water-mediated bridges (grey circles). The high density of interactions in the central region correlates with the predicted structural motif.

**Modified Peptide-HSV-1 gD Complex.**

3. Cytotoxicity assay:

Figure S5: All the peptides were found to be stable throughout the assay. The figure below shows the effect of Native (NP) and modified peptides (F4) on Vero cell viability. Both peptides maintained > 90% cell viability at all tested concentrations, indicating negligible in vitro toxicity.

**Table S3: RAW data for CPE inhibition of Native (NP) and Modified Peptide (F4)**

| Testing | Sample | Concentration (µg/ml) | Average  absorbance | % Protection |
| --- | --- | --- | --- | --- |
| Cycle 1 | **Peptide** | 31.25 | 0.833 | 2.059659 |
|  |  | 62.5 | 0.9 | 11.5767 |
|  |  | 125 | 1.00725 | 26.81108 |
|  |  | 250 | 1.28675 | 66.51278 |
|  | **Acyclovir** | 25 | 1.36225 | 77.23722 |
|  | **Cell Control** | -- | 1.5225 | 100 |
|  | **Virus Control** | -- | 0.8185 | 0 |
| Cycle 2 | **Peptide** | 31.25 | 0.94225 | 4.446743 |
|  |  | 62.5 | 0.9655 | 14.06412 |
|  |  | 125 | 0.98875 | 23.68149 |
|  |  | 250 | 1.0725 | 58.32472 |
|  | **Acyclovir** | 25 | 1.142 | 0.870734 |
|  | **Cell Control** | -- | 1.17325 | 100 |
|  | **Virus Control** | -- | 0.9315 | 0 |
| Cycle 3 | **Peptide** | 31.25 | 0.7975 | 0.014772 |
|  |  | 62.5 | 0.83825 | 0.055522 |
|  |  | 125 | 0.99275 | 0.210022 |
|  |  | 250 | 1.36525 | 0.582522 |
|  | **Acyclovir** | 25 | 1.701 | 0.918272 |
|  | **Cell Control** | -- | 1.95075 | 100 |
|  | **Virus Control** | -- | 1.701 | 0 |
| Cycle 4 | **Peptide** | 31.25 | 0.959 | -0.01464 |
|  |  | 62.5 | 0.98325 | 0.009606 |
|  |  | 125 | 1.12125 | 0.147606 |
|  |  | 250 | 1.566 | 0.592356 |
|  | **Acyclovir** | 25 | 1.77875 | 0.805106 |
|  | **Cell Control** | -- | 1.61 | 100 |
|  | **Virus Control** | -- | 1.77875 | 0 |

Figure S6: Molecular dynamics simulation analysis of the peptide-receptor complexes of a) Native Peptide (NP) and b) Modified Peptide (F4) over the 100ns simulation trajectory. Each panel represents Root Mean Square Fluctuation (RMSF) of the protein and Ligand backbone residues, and Protein ligand contacts.
